# Supplementary material for: Identification of a BAHD Acyltransferase Gene Involved in Plant Growth and Secondary Metabolism in Tea Plants
Source: Plants (Basel). 2022 Sep 22;11(19):2483. doi: 10.3390/plants11192483 (PMC9572432; doi:10.3390/plants11192483)
Supplement: Supplementary file 1 [file plants-11-02483-s001.zip › Additional file S1(FigureS1).pdf]

Identification of a BAHD Acyltransferase Gene Involved in Plant Growth and Secondary Metabolism in Tea Plants

Shirin Aktar [1,2], Peixian Bai [1], Liubin Wang [1], Hanshuo Xun [1], Rui Zhang [1], Liyun Wu [1], Mengdi He [1], Hao Cheng [1], Liyuan Wang [1,*] and Kang Wei [1,*]

1. Key Laboratory of Tea Biology and Resources Utilization, Ministry of Agriculture, National Center for Tea Improvement, Tea Research Institute Chinese Academy of Agricultural Sciences (TRICAAS), Hangzhou 310008, China
2. Graduate School of Chinese Academy of Agricultural Sciences, Beijing 100081, China

* Correspondence: wangly@tricaas.com (L.W.); weikang@tricaas.com (K.W.); Tel.:+86-571-86650575 (L.W.); +86-13656637415 (K.W.)

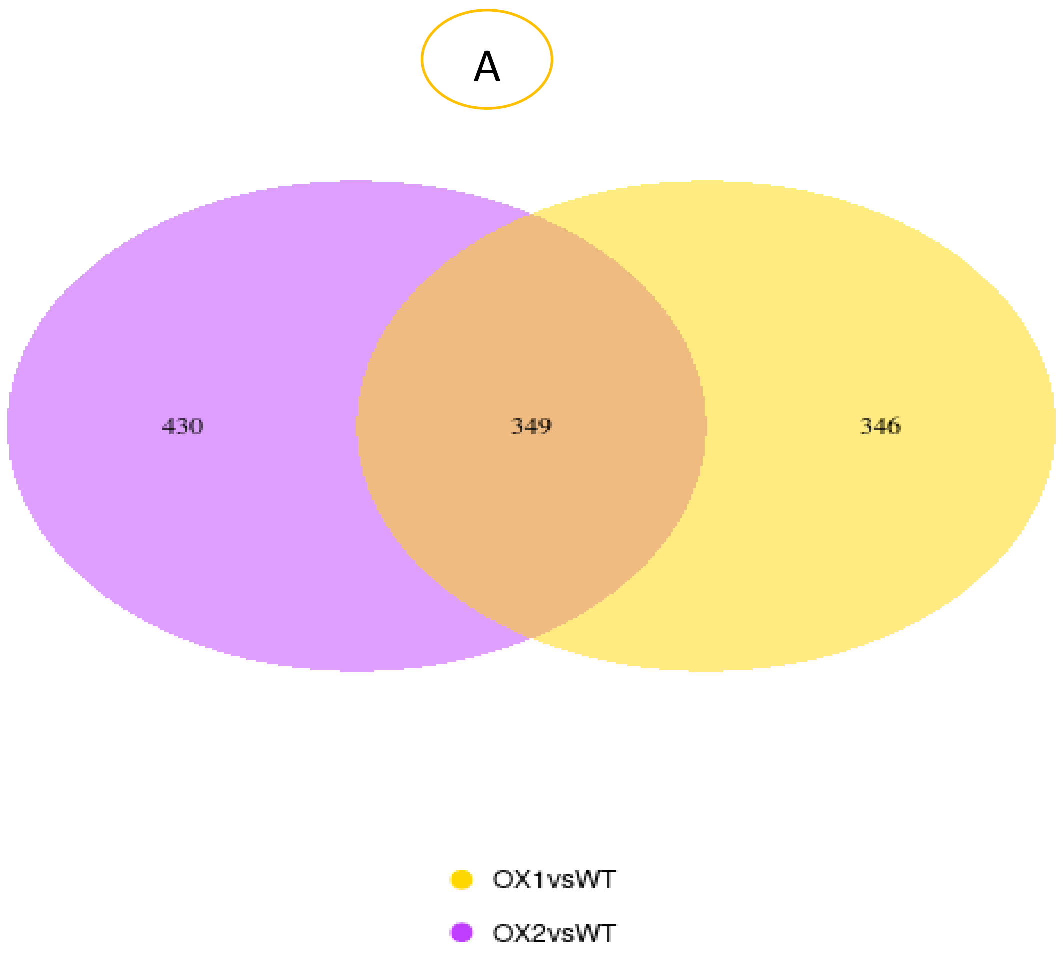
A
430
349
346
OX1vsWT
OX2vsWT

OX1 vs. WT

B

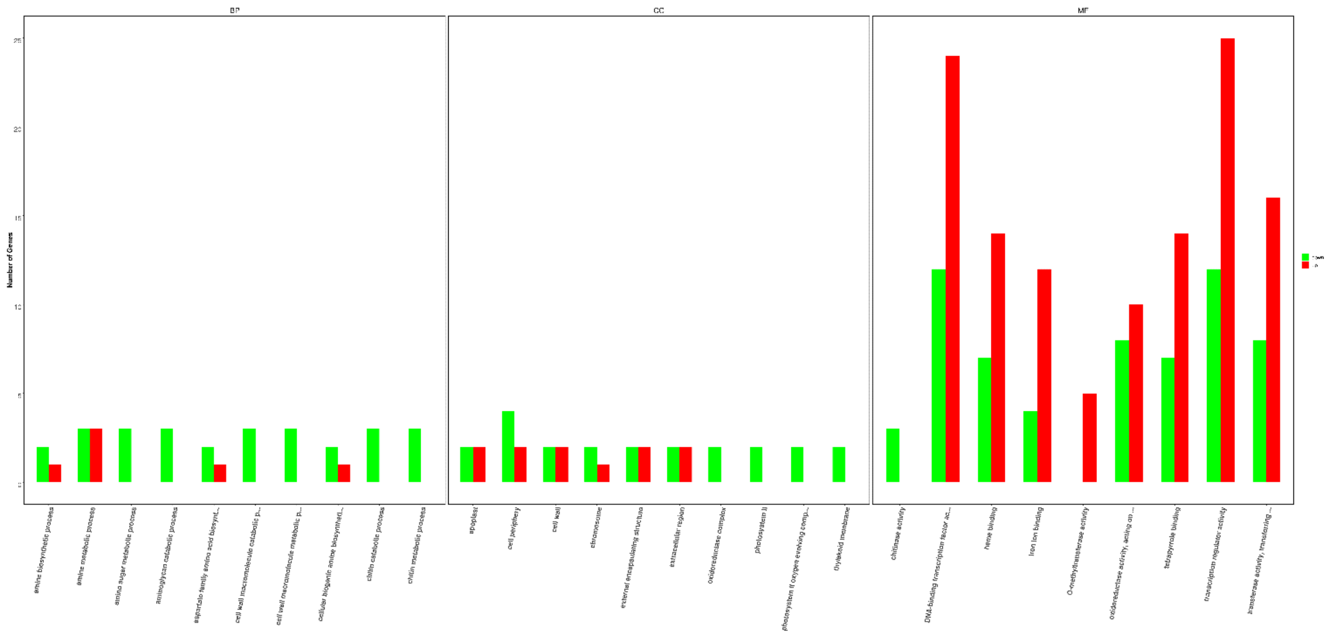

OX2 vs. WT

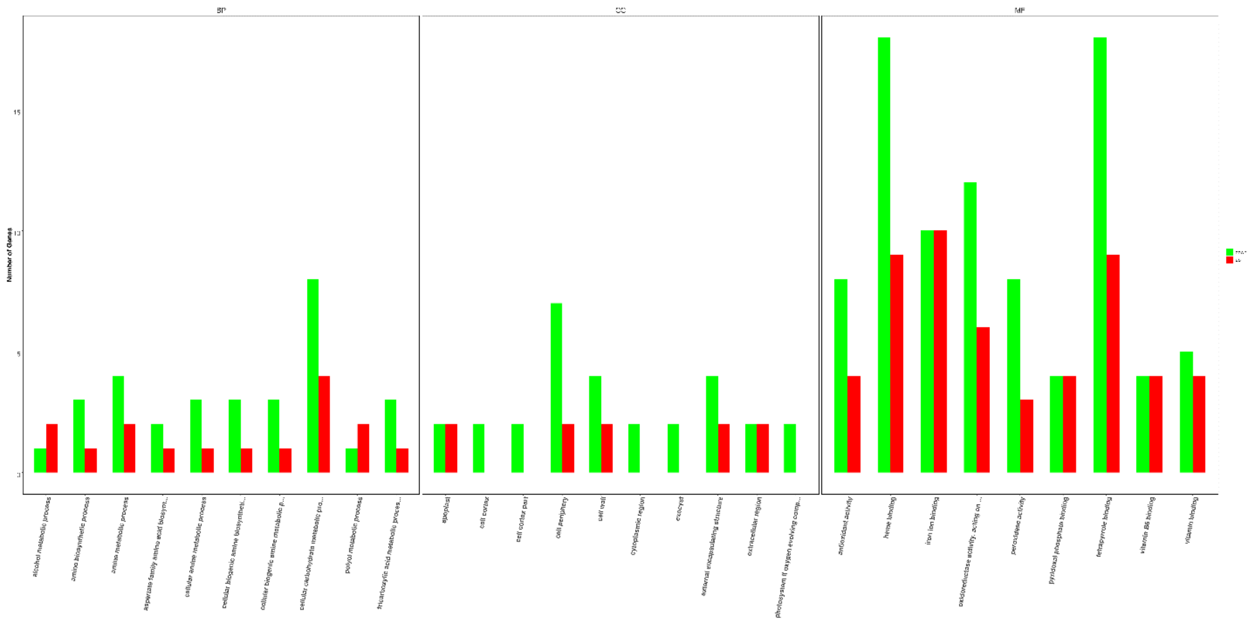

Figure S1. A) The Venn diagram comparison summarizing overlaps in differentially expressed genes among the three comparisons. B) GO assignment and comparison of all DEGs in OX1 vs. WT and OX2 vs. WT. All DEGs in OX1 vs. WT and OX2 vs. WT were annotated in three main categories: biological processes (BP), cellular components (CC) and molecular functions (MF). The left axes indicate the percentage and the number of annotated genes in each category, respectively
