# Supplementary material for: Identification of a BAHD Acyltransferase Gene Involved in Plant Growth and Secondary Metabolism in Tea Plants
Source: Plants (Basel). 2022 Sep 22;11(19):2483. doi: 10.3390/plants11192483 (PMC9572432; doi:10.3390/plants11192483)
Supplement: Supplementary file 1 [file plants-11-02483-s001.zip › Additional file S2(Figure S2 and S3).pdf]

Identification of a BAHD Acyltransferase Gene Involved in Plant Growth and Secondary Metabolism in Tea Plants

Shirin Aktar [1,2], Peixian Bai [1], Liubin Wang [1], Hanshuo Xun [1], Rui Zhang [1], Liyun Wu [1], Mengdi He [1], Hao Cheng [1], Liyuan Wang [1,*] and Kang Wei [1,*]

1. Key Laboratory of Tea Biology and Resources Utilization, Ministry of Agriculture, National Center for Tea Improvement, Tea Research Institute Chinese Academy of Agricultural Sciences (TRICAAS), Hangzhou 310008, China
2. Graduate School of Chinese Academy of Agricultural Sciences, Beijing 100081, China

* Correspondence: wangly@tricaas.com (L.W.); weikang@tricaas.com (K.W.); Tel.:+86-571-86650575 (L.W.); +86-13656637415 (K.W.)

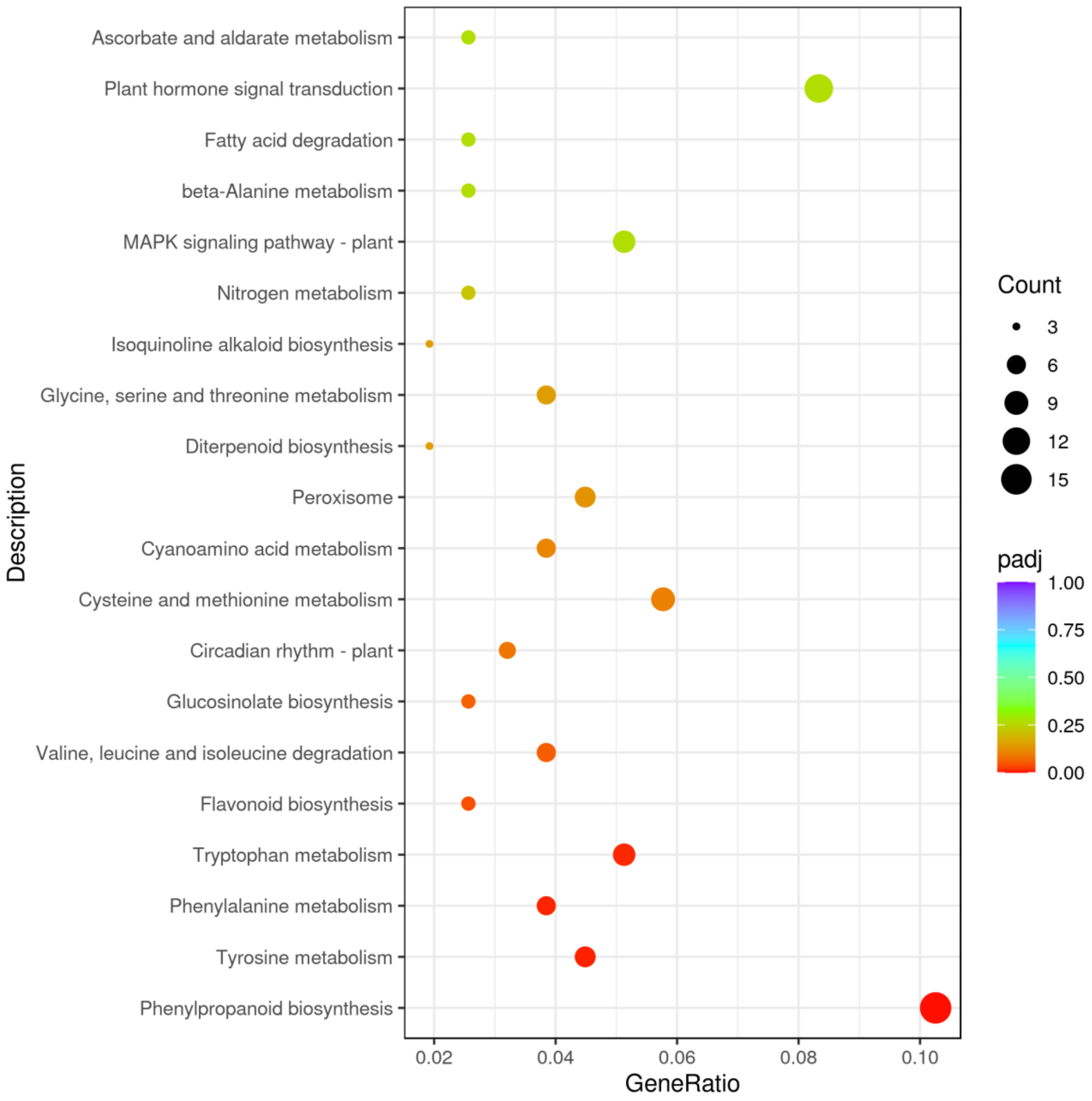

Figure S2. Kyoto Encyclopedia of Genes and Genomes (KEGG) Analysis that Enriched with Significant Metabolic Pathways in OX1 *vs.* WT group under ½ MS media

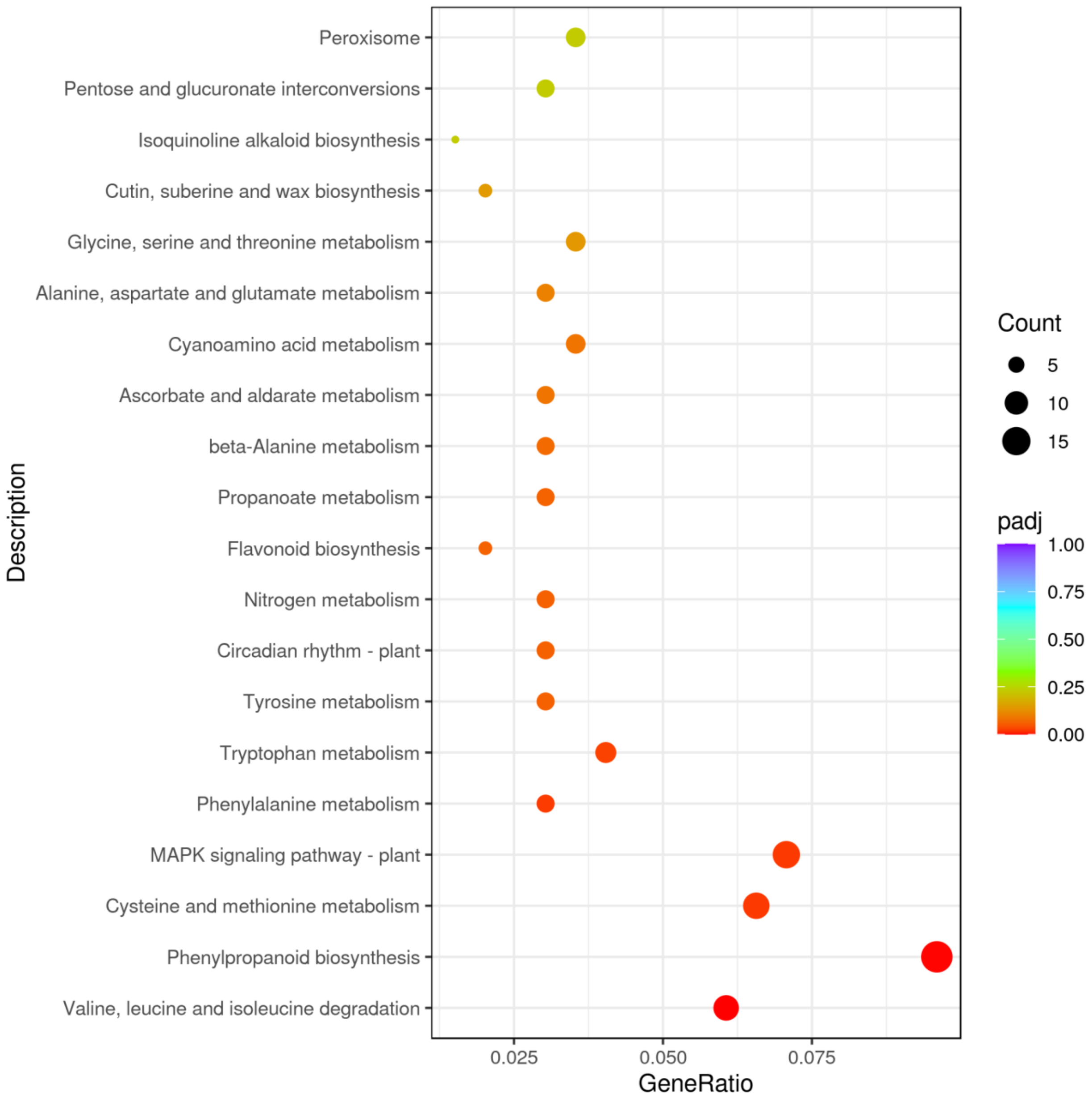

Figure S3. Kyoto Encyclopedia of Genes and Genomes (KEGG) Analysis that Enriched with Significant Metabolic Pathways in OX2 *vs.* WT group under ½ MS media
